# Experiments and Observations Relative to Medicinal Barks

**Published:** 1801-01

**Authors:** W. G. Maton


### *Experiments and Obſervations relative to Medicinal Barks.*
### By Dr. W. G. MATON, F. R. S. &c.

IF it be admitted that the properties of ſubſtances differ according to the different proportions of their ingredients, (and it would ſeem that this principle muſt be admitted in medical as well as in every other natural ſcience) nothing can be more evident than that thoſe moſt allied in compoſition muſt approach moſt nearly to each other with regard to their effects. By purſuing this compariſon alſo we are moſt likely to ſucceed in aſ-

certaining on what ſpecific ingredient, or combination of ingredients, the efficacy of a particular ſubſtance depends; an enquiry which has a powerful claim to the attention of medical profeſſors, but in which, perhaps, leſs progreſs has been made than in any other connected with the healing art. Unaided indeed by the important facts revealed by modern chemiſtry, ſpeculations of this nature *could* not be very ſatisfactory; but even now that the ſtudy of phyſic has received a conſiderable ſhare of the benefits which this ſcience has conferred on the uſeful arts in general, it has ſcarcely begun to emerge from the diſgrace and obſcurity of empiriciſm.

The above remarks will more particularly apply to ſome of thoſe vegetable ſubſtances which are conceived to poſſeſs what is called a *tonic* property. In this diviſion of the materia medica, the Peruvian bark deſervedly holds a diſtinguiſhed place; but whether there are not other ſubſtances (ſome of them indigenous in our iſland) which may in many caſes be exhibited with equal utility, is ſtill a queſtion with many eminent members of the medical profeſſion, and muſt continue to be ſo until chemical analyſis has diſcovered the preciſe compoſition of this celebrated bark, compared with that of other vegetable productions which contend for equal reputation. Some experiments connected with ſuch an enquiry, it is my object to bring forward in the preſent paper; but they form ſo inconſiderable a part of the ſeries neceſſary to the deciſion of the important queſtions above alluded to, that I bring them forward with much heſitation, and hope that they will be regarded rather as hints to perſons who have great leiſure for the proſecution of theſe curious inveſtigations, than as an addition of much value to the ſtock of medical facts.

By no analyſis of the Peruvian bark hitherto publiſhed,* has it appeared that the principle denominated by the French chemiſts *tannin*, was diſcoverable in that ſubſtance; indeed, we do not any where find this principle referred to prior to the experiments of Seguin, which had in view the elucidation of the *rationale* of tanning, but which, like many diſcoveries at firſt connected apparently with only one particular art, may hereafter throw a new light on other ſubjects of much higher importance.† As this point did not appear to have been touched upon,

* Since the above was written, a recent number of the *Annales de Chimie* has announced that Dr. Woſtring, a phyſician of Norcop, in Sweden, has aſcertained the proportion of tannin contained in the ſeveral ſpecies of Cinchona. We are not informed, however, of the requiſite particulars relating to thoſe experiments.

† See "*Rapport au Comite de Salut public, ſur les nouveaux moyens de tanner les cuirs, proposés par le Cit. Armand Seguin. 3. Brumaire, l'an 3 de la Rep.*" (1795.)

upon, it was the firſt which I propoſed to myſelf to inveſtigate, and the following is the reſult of my experiments with the Peruvian, Caſcarilla, Oak and Elm barks, and Gall-nuts.† Half an ounce of each, very finely powdered, was infuſed two hours in half a pound of ſpring water, care being taken to ſtir the mixture very frequently, and at the end of this time, half an ounce of a ſolution of iſinglaſs (made by diſſolving half an ounce of the latter in one pound of water) was poured into the ſtrained infuſion. It was obſervable that the liquor ſtrained from the Peruvian bark was by far the cleareſt, and that of the Caſcarilla the moſt turbid. The elm bark contained ſo large a quantity of viſcid, yellowiſh mucilage, that it was with the utmoſt difficulty the infuſion could be ſtrained, and it ſtained the paper, &c. through which it was filtered of a reddiſh brown colour; that left by the other barks was pretty eaſily waſhed off by means of pure water only, but the colour of the elm yielded only to the infuſion of galls, which effectually eradicated alſo the others. Another circumſtance proper to be mentioned was, that on a certain quantity of the infuſion of galls being accidentally poured into ſome of the infuſion of bark, the colouring matter of the latter was ſpeedily precipitated, and formed a pinkiſh cloud at the bottom of the veſſel; a ſimilar effect was produced on the infuſion of Caſcarilla, but not on the other infuſions.—No change being produced on half an ounce of the liquor of the Peruvian bark by half an ounce of the ſolution of iſinglaſs, the quantity of the former was doubled; in a few minutes a very ſlight flocculent appearance was perceivable, which however, during half an hour, did not form more than a cloud. Another half ounce of the infuſion was added, but at the end of forty hours the precipitate was ſcanty, and weighed only a quarter of a grain. When dry, this precipitate became tough, ſomewhat tranſparent, and of the colour of carmine.—2. One ounce of the infuſion of Caſcarilla was not affected by the ſolution of iſinglaſs; but ſoon after another half ounce ha[illegible] been added thin flocculi collected pretty faſt, yet it was not until forty hours had elapſed that the precipitate could be collected, and it was then pretty compact, brown, and weighed 1 ¼ grain.—3. Equal quantities (half an ounce) of the infuſion of oak and ſolution of iſinglaſs yielded a precipitate immediately, the mixture having firſt aſſumed a curdled appear-

---

† Great pains were taken to procure the beſt kinds of theſe ſeveral ſubſtances; the Peruvian bark which I employed was the red ſort, and obtained, as well as the Caſcarilla, at Apothecaries Hall.

ance, which was found to confift of an infinite number of fine flocculi coagulating quickly, and in a moft beautiful manner, into a dark brown fediment. I could not doubt that, at leaft with refpect to this bark, I had obtained the neutralized tannin of Seguin, whofe defcription of the precipitate which he had procured from the fame bark, correfponded exactly with mine. The precipitate did not adhere to the fingers even when moift, but was readily fqueezed into a compact mafs, which became extremely hard and brittle, and of a blackifh brown colour; it weighed when quite dry, rather more than four grains. Half a drachm of folution of ifinglafs was added to the liquor poured off from the precipitate, but there was no appearance of flocculi; the ifinglafs, at the end of forty hours, had funk to the bottom of the veffel almoft pure. — 4. The ftrained infufion of the elm bark exhibited a flight flocculency as foon as the liquid ifinglafs was added. After the fpace of forty hours, a confiderable precipitate was formed, which, when dried, weighed fix grains, but was not very compact, the colour reddifh brown.—5. The infufion of galls, after having been treated fimilarly to that of the oak bark changed inftantly from a light greenifh brown to a milky white colour; the mixture was now nearly taftelefs. At length a white precipitate was formed, which, when weighed, was found to amount to the quantity of eleven grains and a half, and had become a very hard but friable mafs. To the liquor poured from this precipitate I added half an ounce more of the folution of ifinglafs. The mixture affumed the appearance of whey, and yielded another precipitate, not fo ftrongly formed as the firft, but weighing five grains and a half, and its colour was more inclined to yellow. Half a drachm of folution being added to this laft liquor, and the whole quantity allowed to ftand forty hours, nothing but ifinglafs was to be feen at the bottom of the veffel, and the liquor had begun to be more of a green colour, which gradually deepened towards the furface. The laft mentioned circumftance was obfervable with refpect alfo to the Peruvian and the Elm bark infufions (ftrained from the precipitates) after they had ftood the fame length of time.

It was now neceffary to make an accurate examination of the above mentioned precipitates, and to afcertain whether they were all really the combination of the gelatine with the tanning principle of the refpective barks. But I was defirous of difcovering previoufly, the relations of the feveral infufions to one another, with refpect to colour, after an admixture of each with a certain quantity of a folution of iron; and of comparing the appearance of the infufions which contained all the ingredients in in ſolution, with that of the liquor from which the precipitates had been obtained; for I was not convinced that a portion of gallic acid could not have gone down with the *tannin* in conſequence of being alſo neutralized by the gelatine. This indeed was but a rough mode of aſcertaining the portion of gallic acid, but it ſeemed to be worthy of trial. Half a drachm of a ſolution of the green ſulph. of iron in diſtilled water was added to half an ounce of each of the infuſions which had formed precipitates, and to a like quantity of the ſame not deprived of their *tannin*. The colours did not prove more intenſe in the laſt mentioned infuſions than in the firſt. There was a ſtriking difference, however, in the quantity of gallic acid manifeſted by the ſeveral barks, the Oak aſſuming a deep inky purple colour, as did alſo the Peruvian; whereas the Caſcarilla was but ſlightly altered, in compariſon with the others. The elm infuſion acquired a browniſh purple tint, which was pretty deep, the galls a purple more inclined to red.

It was plain that all the barks had been infuſed long enough to evolve at leaſt a part of their gallic acid, and I was of opinion that the above experiment was not nice enough to determine whether any had gone down in the precipitates; this could be decided only by an accurate analyſis of the precipitates themſelves. That gallic acid has an affinity to animal gluten is ſufficiently evident from the memoir of Cit. Seguin, who thinks that it has its effect in the proceſs of tanning, though whether detrimental or otherwiſe he does not appear to have proved.*

Why a longer portion of time has been preſcribed for the infuſion of barks, in order to extract the gallic acid, than has been thought neceſſary for the extraction of tannin, was another point concerning which I was by no means ſatiſfied, and I began to doubt whether by an infuſion of only two hours I had procured as much of the *latter* as the barks might really contain, eſpecially as ſome ſeemed to give it out to the water more ſlowly than others. It was likewiſe uncertain whether the proportion of the bark to the menſtruum was ſuch as was moſt favourable to the complete evolution of all the ſoluble parts of that ſubſtance. It appeared therefore proper to obſerve the effects of the ſolution of iſinglaſs on an infuſion of longer ſtanding than has been thought ſufficient in pharmaceutical proceſſes;† and alſo

* Mr. Biggin, who (though ſo well known before as a gentleman of talents and zeal in the proſecution of philoſophical experiments) juſtly deſerves diſtinguiſhed mention for his examination of ſubſtances moſt advantageous in tanning, has informed me that he is convinced of the *injuriouſneſs* of this acid.

† See Lewis's *Materia Medica* (laſt edit. by Dr. Aikin) article *Cortex Peruvianus*.

alſo to try at what point of time, or by what means, the liquor might be known to hold all the *tannin* that the bark contained.

[ To be continued. ]

*Craven Street, Nov.* 20, 1800.

